# Periodic Manual Algorithm Updates and Generalizability: A Developer’s Response. Comment on “Evaluation of Four Artificial Intelligence–Assisted Self-Diagnosis Apps on Three Diagnoses: Two-Year Follow-Up Study”

**DOI:** 10.2196/26514

**Published:** 2021-06-16

**Authors:** Stephen Gilbert, Matthew Fenech, Anisa Idris, Ewelina Türk

**Affiliations:** 1 Ada Health Berlin Germany

**Keywords:** artificial intelligence, machine learning, mobile apps, medical diagnosis, mHealth, symptom assessment

We have several comments on the recent publication of Ćirković [1], in which repeated testing of four symptom assessment applications with clinical vignettes was carried out to look for “hints of ‘non-locked learning algorithms’.” As the developer of one of the symptom assessment applications studied by Ćirković [1], we are supportive of studies evaluating app performance; however, there are important limitations in the methodology of this study.

Most importantly, the methodology used in this study is not capable of addressing its main objective. The approach used to look for evidence of nonlocked algorithms was the quantification of differences in performance using 3 ophthalmology vignettes, first in 2018, then in 2020. This methodology, although highly limited due to the use of only 3 vignettes in one medical specialism, could be used to detect changes in app performance over time. It, however, cannot be used to distinguish between nonlocked algorithms and the manual updating of apps’ medical intelligence, through the normal process of the manual release of updated app versions. Medical device regulations and quality system requirements provide standard mechanisms through which apps can be further developed, validated, and released as updated versions. The manual of medical knowledge in this manner has been acknowledged by the manufacturers of all the apps studied by Ćirković [1]. In response to previous independent vignettes studies [2,3], spokespeople for Your.MD and Babylon stated that they update their medical knowledge periodically, and this is also clear on Buoy’s website. In Gilbert et al [4], the Ada app is described as having a knowledge base “built and reviewed by medical doctors in a curated process of knowledge integration from medical literature. It is being expanded continuously following this standardized process.”

As is acknowledged in the limitations listed in Ćirković’s work [1], the study used vignettes designed, entered, and with results adjudicated by a single clinician. This could result in bias and a narrow type of case. It is also acknowledged that 3 vignettes represent a small sample size for a vignettes study and that “standardized and transparent procedures” are needed for symptom assessment app–vignettes studies. We recently published a 200-vignette assessment of symptom assessment applications [4], including those studied by Ćirković [1], which used standardized and transparent procedures, including the separation of vignette design, entered and with results adjudication. It is our view that the effect of the limitations described by Ćirković [1], together with only including ophthalmological cases, is that the accuracy results reported have limited generalizability or repeatability. Our own internal validation testing shows an improvement in Ada's medical intelligence in all-condition top-3 suggestion accuracy (also known as M3, as defined by Miller et al [5]) of 4.8% between 2018 and 2020. We take account of all performance feedback we receive, and incorporate this, when judged appropriate by our medical knowledge experts, into updates of our app, through periodic releases of locked versions of our app.
